# Population structure of three *Psammodromus* species in the Iberian Peninsula

**DOI:** 10.7717/peerj.994

**Published:** 2015-06-04

**Authors:** Jose L. Horreo, Patrick Fitze

**Affiliations:** 1 Department of Ecology and Evolution, University of Lausanne, Biophore, Lausanne, Switzerland; 2 Instituto Pirenaico de Ecología (IPE-CSIC), Jaca, Spain; 3 Fundación Araid, Zaragoza, Spain; 4 Department of Biodiversity and Evolutionary Biology, Museo Nacional de Ciencias Naturales (MNCN-CSIC), Madrid, Spain

**Keywords:** Lizard, Conservation, Migration, Roads, *Psammodromus edwardsianus*, *Psammodromus hispanicus*, *Psammodromus occidentalis*

## Abstract

The knowledge of a species’ population structure is essential for the development of adequate conservation actions as well as for the understanding of its evolution. The population structure is unknown in all species of the Genus *Psammodromus*, including the Western Sand Racer (*Psammodromus occidentalis*; a recently described species), the Edward’s Sand Racer (*P. edwardsianus*) and the Spanish Sand Racer (*P. hispanicus*). In this article, the genetic variability and population structure of *Psammodromus edwardsianus*, *P. hispanicus*, and *P. occidentalis* were studied in the Iberian Peninsula covering their natural geographic distribution. Mitochondrial DNA showed genetically different units in all species with higher genetic variability in their southern populations (latitudinal variation). Genetic differentiation was different among species and contrasted to those of species with similar characteristics. Our results therefore highlight the importance of species-specific studies analysing population structure.

## Introduction

The knowledge of the population structure of a species is basic for its efficient conservation and management as well as for the understanding of subjacent evolutionary processes implicated on it (Millar & Libby, 1991). Differences in genetic diversity can have substantial effects on ecological processes (Hughes et al., 2008) as well as on the success of conservation; thus, studies analysing the congruence of population structure among similar species are required to understand their predictability. Genetic studies are of particular interest for evaluating the conservation status of newly described species’ in a quick and robust way because genetic differentiation is a crucial factor shaping a species’ population structure (Avise, 2000). Moreover, genetic diversity within local populations is essential to ensure its adaptive and evolutionary flexibility and the long-term survival of the species (Templeton, 1991). For this reason, the maintenance of genetic diversity is one of the most important aims of species conservation (Barrett & Kohn, 1991), and the identification of hotspots of genetic diversity is one of the first steps in the development of management actions (Fleishman et al., 2002).

The three species of the Genus *Psammodromus P. occidentalis, P. edwardsianus* and *P. hispanicus* (the Western Sand Racer, the Edward’s Sand Racer, and the Spanish Sand Racer, respectively) have been recently described as new lizard species given their old divergence and their genetic, phenotypic, and ecological niche differences (Fitze et al., 2012). *P. occidentalis* diverged 8.3 (2.9–14.7) Mya from the ancestor of *P. edwardsianus* and *P. hispanicus*, and the latter diverged 4.8 (1.5–8.7) Mya (Fitze et al., 2011). The natural distribution of these three species ranges from southern Spain to southern France and ecological niche modelling showed that suitable habitat of *P. occidentalis* and *P. edwardsianus* overlap over vast areas, while the other species, *P*. *hispanicus,* inhabits an ecological niche that overlaps marginally with the other two lineages (Fitze et al., 2011). Phylogenetic analyses indicate that *P. occidentalis* diverged from the ancestor of *P. edwardsianus* and *P. hispanicus*, and the observed niche conservatism, as well as the current geographic distribution, suggest that speciation happened in allopatry. In contrast, *P. edwardsianus* and *P. hispanicus* speciated during the Messinian salinity crisis during which major geologic and climatic changes occurred. The date of the split together with the marginal niche overlap suggests that niche divergence was responsible for the speciation of these last two species. Thus two distinct and temporally separated processes may have probably led to the observed speciation events.

Some phylogenetic and phylogeographic studies have been previously done in different *Psammodromus* species (Carranza et al., 2006; Fitze et al., 2011; Verdú-Ricoy et al., 2010). Nevertheless, little is known about any of the *Psammodromus* species’ population structure. Moreover, a recent article suggests that sexually selected traits could prevent reproduction and gene flow at secondary contact zones among them, which may reinforce their isolation (San-Jose, Gonzalez-Jimena & Fitze, 2012) and would be congruent with patterns observed in species with similar characteristics (e.g., *Psammodromus algirus*, *Sceloporus occidentalis, Aspidoscelis hyperythra*; Telleria et al., 2011; Brehme et al., 2013). In order to unravel the population structure, conservation status and their congruence among species, the population genetics of three recently described species (*P. edwardsianus*, *P. hispanicus*, and *P. occidentalis*) was studied, using mitochondrial DNA and a sampling covering the biggest area of their natural geographic distribution. Results will be very useful, for conservation and management issues, among other reasons.

## Materials and Methods

### DNA samples

**Figure 1:**
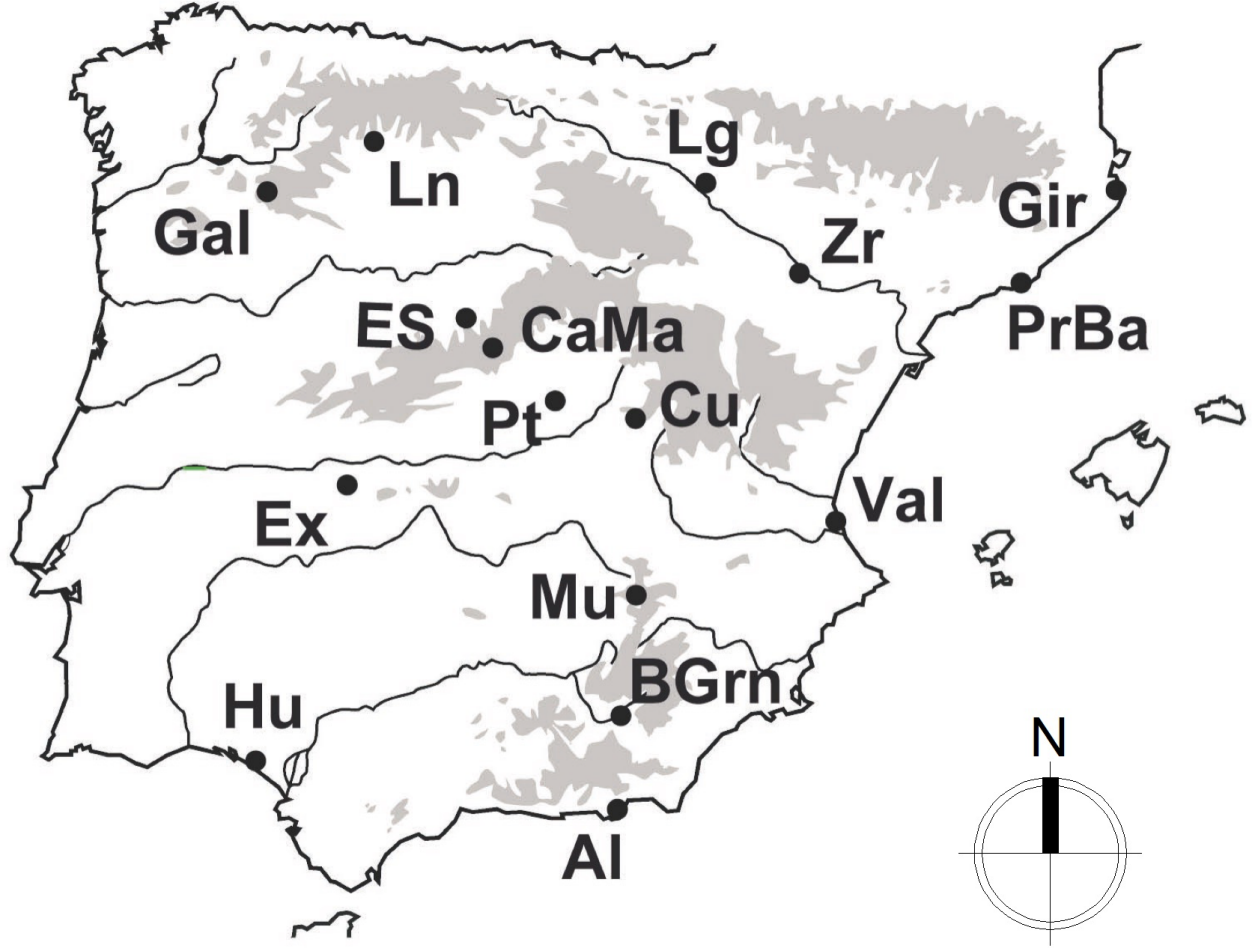
Sampling distribution of *Psammodromus edwardsianus*, *P. hispanicus* and *P. occidentalis* in the Iberian Peninsula.

Following the recent nomenclature described by Fitze et al. (2012), a total of 247 individuals from *Psammodromus hispanicus* (*n* = 69), *P. edwardsianus* (*n* = 135) and *P. occidentalis* (*n* = 43) were used in this study. The study employs all of the currently available genetic data and it includes three of the six described species of the Genus (note: for the other species of this genus no intensive population sampling has been conducted to date). Cytochrome B (cytB) sequences of the studied species (the only marker with enough sample sizes for this study) were obtained from GenBank (for accession numbers and original procedures, see Fitze et al., 2011). All used sequences stem from specimens collected in the same year, under standardized conditions and over the largest part of their natural geographic distribution (Fig. 1), which guarantees high comparability. Sequences were visualized, edited, and aligned employing BioEdit (Hall, 1999) and the ClustalW algorithm (Thompson, Higgins & Gibson, 1994).

### Population genetic analyses

All populations with a sample size of at least nine individuals were included in the sequence datasets (Table 1). The only exception was the population HuEx (*n* = 8), where two populations from neighbouring provinces have been joined (Hu, *n* = 5 and Ex, *n* = 3) because unfortunately not any of the sampled southern *P. occidentalis* populations consisted of more than 8 captured individuals.

**Table 1:** Genetic variability within populations and species. Shown are sample size (*n*), haplotype diversity (*h*) and nucleotide diversity (*π*), the latter two are measured in %. Tajima’s *D* and Fu’s *Fs* test for population expansion. (A) *Psammodromus edwardsianus* (B) *P. hispanicus*, and (C) *P. occidentalis*.

	n	h	*π*	*D*	*Fs*
**(A)**					
Val	22	70.60	0.482	1.182	1.199
BGrn	15	62.90	0.566	−0.281	0.440
Al	14	38.46	0.221	−1.278	−0.314
PrBa	24	29.98	0.116	0.138	0.268
Gir	27	0.00	0.000	0.000	0.000
Zr	18	11.11	0.089	−0.507*	0.070
Cu	15	73.33	0.382	0.096	−0.443
**(B)**					
Mu	9	55.60	0.848	−0.804	1.919
Lg	23	16.60	0.067	−0.662	−0.213
ES	28	26.45	0.110	−0.972	−1.090
Pt	9	41.67	0.714	−1.797*	1.520
**(C)**					
CaMa	13	28.20	0.113	−0.274	0.240
Ln	9	0.00	0.000	0.000	0.000
Gal	13	0.00	0.000	0.000	0.000
HuEx	8	85.70	0.047	1.131	2.797

**Notes.**

* *P* < 0.05.

Genetic variability, measured as haplotype (h) and nucleotide (*π*) diversity, as well as genetic differentiation among populations, measured as *F*_ST_ (and their corresponding *P*-values), were calculated with Arlequin v3.11 software (Excoffier & Lischer, 2010). The same software was employed for the Analysis of Molecular Variance (AMOVA; 1,000 permutations) with the total *Psammodromus* dataset and for searching signals of population expansions in the analysed populations testing deviation from equilibrium expectations with Tajima’s *D* (Tajima, 1989) and Fu’s *Fs* (Fu, 1997) neutrality tests based on an infinite-site model without recombination.

Isolation by distance (IBD) was studied with GENEPOP (Rousset, 2008) employing 10,000 permutations and a Mantel test to investigate whether *F*_ST_ values transformed as *F*_ST_/(1 − *F*_ST_) are linearly linked with the logarithm of the distance (in ln[km]) among the sampled populations, as predicted by a two-dimensional migration model (Rousset, 1997). In *P. occidentalis* IBD was studied without the HuEx population because HuEx consists of two populations.

## Results

Genetic diversity varied among populations in all the three species (Table 1). The haplotype diversity ranged from 0 to 71% in *Psammodromus edwardsianus*, from 0 to 86% in *P. occidentalis* and from 17 to 56% in *P. hispanicus*. *P. hispanicus* was the only species without monomorphic populations. Nucleotide diversity ranged from 0.000 to 0.566 in *P. edwardsianus*, from 0.067 to 0.848 in *P. hispanicus* and from 0.000 to 0.113 in *P. occidentalis*. Neither tests (Tajima’s *D* and Fu’s *Fs*; Table 1) provided evidence of population expansion for most populations, except for Zr in *P. edwardsianus* and Pt in *P. hispanicus* using Tajima’s *D*.

AMOVA (Table 2) showed highly significant genetic differentiation among species as well as among populations within species (*P* < 0.001 in both cases). *F*_ST_ values (Table 3) showed significant genetic differentiation (*P* < 0.05) among almost all populations of *P. edwardsianus* except between the two northernmost populations (population pairs PrBa-Zr and Zr-Gir). In *P. occidentalis* three of the six population differences were significant (Table 3C) and among the northern populations no significant differentiation existed. In *P. hispanicus*, MU significantly differed from the other populations, showing that no significant differentiation existed among northern populations.

**Table 2:** AMOVA results considering each of the three studied species (*Psammdoromus edwardsianus*, *P. hispanicus* and *P. occidentalis*) and their populations.

Source of variation	Variance components	Percentage of variation	*P*-value
**Among species**	10.75834	88.7	0.000
**Among populations within species**	0.86622	7.14	0.000
**Within populations**	0.50373	4.15	0.000

**Table 3:** Genetic differentiation (measured with *F*_ST_ values), among the different sampled populations of (A) *P. edwardsianus*, (B) *P. hispanicus* and (C) *P. occidentalis*.

(A)	Val	BGrn	Al	PrBa	Gir	Zr	Cu
**Val**	–						
**BGrn**	0.76^*^	–					
**Al**	0.78^*^	0.82^*^	–				
**PrBa**	0.39^*^	0.83^*^	0.89^*^	–			
**Gir**	0.46^*^	0.89^*^	0.94^*^	0.14^*^	–		
**Zr**	0.37^*^	0.82^*^	0.89^*^	0.07	0.02	–	
**Cu**	0.34^*^	0.76^*^	0.80^*^	0.22^*^	0.29^*^	0.19^*^	–

(B)	Mu	Lg	ES	Pt
**Mu**	–			
**Lg**	0.57^*^	–		
**ES**	0.56^*^	0.04	–	
**Pt**	0.22^*^	0.09	0.09	–

(C)	CaMa	Ln	Gal	HuEx
**CaMa**	–			
**Ln**	0.04	–		
**Gal**	0.08	0.00	–	
**HuEx**	0.63^*^	0.57^*^	0.64^*^	–

**Notes.**

* *P* < 0.05

Isolation by distance (IBD) tests resulted highly significant (*P* < 0.01) in *P. edwardsianus*. In *P. hispanicus* and *P. occidentalis*, no significant IBD was found.

## Discussion

This is the first article describing the population structure of a *Psammodromus* species. Phylogenetic studies have been previously conducted for the three studied species (*Psammodromus edwardsianus*, *P. hispanicus*, and *P. occidentalis*; Fitze et al., 2011) and in one of the other species of the same Genus (*P. algirus*) phylogeographic studies exist (Carranza et al., 2006; Verdú-Ricoy et al., 2010). Despite the inherent limitations of mitochondrial DNA and the limited number of samples available, our results showed different population structures depending on species, but all three species exhibited significant genetic differences among them as well as a similar latitudinal (i.e., north–south) genetic structure.

In *P*. *edwardsianus*, northern populations (Gir, PrBa, and Zr) had lower genetic variability than southern populations (Table 1A), the former containing one monomorphic population (Gir). These northern populations, located in the Ebro Valley, also form a genetic separate unit consisting of populations without significant genetic differentiation between them (except between PrBa and Gi). Isolation by distance (IBD) was present in this species.

For *P. hispanicus*, IBD was not significant, no-genetic differentiation existed among populations, and genetic variability was high, suggesting that their populations are connected. The only differentiated population was the MU population. Genetic variability in this species increased with latitude, which is in line with the higher haplotype variability of the southern populations compared to the northern ones (Table 1B).

*P. occidentalis* did not show significant IBD and genetic differences were found among most population pairs, except among the HuEx and the other populations. The two northern populations were monomorphic, while the southern populations had high genetic variability (Table 1C).

As observed in a diverse range of species, the here studied lizards have genetically isolated populations, which requires special management actions because, among others, they are more sensitive to environmental changes (Vucetich & Waite, 2003). The isolation of the populations cannot be explained by any of the big geographic barriers of the Iberian Peninsula (except the Ebro Valley in *P. edwardsianus*), nor by anthropogenic infrastructures such as roads. Big highways or heavy traffic roads determine the movement of other lizard species and small and big mammals (Telleria et al., 2011; Brehme et al., 2013; Franz et al., 2010; Frantz et al., 2012). The populations of the here studied species are separated by several major high-ways (at least 4 lanes). Nevertheless there existed relatively low genetic differentiation in *P. hispanicus* and *P. occidentalis* (only 1 population differed from the other populations). Our results suggest that roads do not determine their population structure, at least at global scale, suggesting that road networks affecting species gene flow might be species-specific (Garcia-Gonzalez et al., 2012). Additionally, these isolated populations did not seem to follow a general geographic central-marginal pattern of genetic diversity, where higher genetic diversity would exist in the core populations compared to the populations at the borders of the species’ geographic distribution (Eckert, Samis & Lougheed, 2008). However, fine scale sampling is required to underpin this hypothesis. Nevertheless, all the three studied *Psammodromus* species followed a latitudinal gradient of genetic diversity, with reduced diversity in northern populations, which is in line with the general patterns described for vertebrates (Adams & Hadly, 2013). The detected low genetic population differentiation in *P. hispanicus P. occidentalis* might exist due to the relatively low number of sampled individuals (Frantz et al., 2012) or due to the use of mitochondrial DNA only, which could result in incomplete genetic information, and it would therefore be advantageous to corroborate the observed patterns using nuclear DNA and bigger sample sizes. Nevertheless, the sample size and the mitochondrial data allowed detecting a latitudinal gradient of genetic diversity in all three species, showing that broad and/or ancient, but may be not fine or recent sources of genetic diversity can be detected.

As commented above, IBD was only significant in *P. edwardsianus*, but not in the other two species. The lack of IBD might be explained by the species distribution and the geographic sampling. First, the geographic distribution of *P. hispanicus* covers mainly the northern part of the southern Peninsula, and only one population was sampled on the southern Peninsula. Second, in none of the sampled southern populations of *P. occidentalis* could we obtain enough individuals to test for population differentiation, despite several months of fieldwork in this area. To test whether the lack of southern populations may have affected the results, the IBD analysis was repeated in *P. edwardsianus* using a subset containing no southern populations (BGrn, and Al). The subset rendered drastic differences since IBD was not longer significant (*P* = 0.192). This suggests that in *P. hispanicus* and *P. occidentalis* IBD results might be affected by the sampling. Thus, the here observed absence of IBD in the two species requires careful interpretation and its presence cannot be discarded.

In sum, mitochondrial DNA showed that all three studied *Psammodromus* species are distributed along the Iberian Peninsula in fragmented units with higher genetic variability in southern populations (latitudinal variation). In conservation terms, isolated populations (the Ebro valley genetic unit in *P. edwardsianus*, the MU population in *P. hispanicus* and HuEx in *P. occidentalis*) as well as those with low genetic variability (the northern populations of all the three species) require specific measures and have to be specially managed together with the more variable populations, because a balanced and dynamic conservation strategy is needed (Mcdonald-Madden, BPW & Possingham, 2008). In addition to this, the genetic differences among populations described here suggest that road infrastructures do not affect the species’ population structure at global scale, what contrasts to other lizard species of similar characteristics (Telleria et al., 2011; Brehme et al., 2013). This finding is in line with a study in amphibians (Garcia-Gonzalez et al., 2012) and highlights the importance of species-specific studies regarding this issue.

## Supplemental Information

10.7717/peerj.994/supp-1 Supplemental Information 1 *Psammodromus edwarsianus* Cytochrome B dataset Psammodromus edwarsianus Cytochrome B sequences dataset in fasta format.

10.7717/peerj.994/supp-2 Supplemental Information 2 *Psammodromus occidentalis* Cytochrome B dataset Psammodromus occidentalis Cytochrome B sequences dataset in fasta format.

10.7717/peerj.994/supp-3 Supplemental Information 3 *Psammodromus hispanicus* Cytochrome B dataset Psammodromus hispanicus Cytochrome B sequences dataset in fasta format.
